# Blood Transcriptome Analysis Reveals Immune Characteristics of Captive Forest Musk Deer (*Moschus berezovskii*) at High Altitude in Bianba County, Tibet

**DOI:** 10.3390/ani15233501

**Published:** 2025-12-04

**Authors:** Lei Chen, Xuxin Li, Zhoulong Chen, Jin Bai, Yanni Zhao, Maoyuan Gan, Wenjingyi Chang, Jieyao Cai, Xiuyue Zhang

**Affiliations:** 1 Key Laboratory of Bio-Resources and Eco-Environment, Ministry of Education, College of Life Sciences, Sichuan University, Chengdu 610064, China; geminist_cl@163.com (L.C.); chenzhoulong1010@163.com (Z.C.); caijieyao0310@163.com (J.C.); 2 Zhangzhou Pien Tze Huang Pharmaceutical Co., Ltd., Zhangzhou 363099, China; lxx@zzpzh.com; 3 Bianba County Musk Deer Breeding Base, Changdu 855419, China; 4 The Conservation of Endangered Wildlife Key Laboratory of Sichuan Province, College of Life Sciences, Sichuan University, Chengdu 610064, China

**Keywords:** forest musk deer, high-altitude adaptation, seasonal variation, immune adaptation, metabolic reprogramming, transcriptomics

## Abstract

The hypoxic, cold, and high-ultraviolet radiation environments at high altitude pose severe challenges to mammalian immune and metabolic systems. However, little is known about how captive forest musk deer adapt to high-altitude environments and their seasonal variations. This study analyzed peripheral blood transcriptomes of 33 captive forest musk deer (*Moschus berezovskii*) at high altitude (~3900 m) and low altitude (~1450 m) during autumn-winter and spring-summer seasons. Results revealed comprehensive immune suppression in the high-altitude group during autumn-winter (downregulation of complement system *CFB*/*C2*/*C3*, interferon pathway genes including *FLT3*, with only natural killer (NK) cell *PRKCQ* upregulated), coupled with energy-conserving metabolic reprogramming (altered carbohydrate metabolism, inhibited lipid synthesis, fat mobilization, suppressed protein degradation). During spring-summer, neutrophil antimicrobial responses (*SLPI*/*NCF1*/*ELANE*) and humoral immunity (B cell differentiation genes *PAX5*/*RUNX1*; class-switch enzyme *AICDA*) partially recovered while cellular immunity (*IL15*/*B2M*) remained suppressed, accompanied by enhanced anabolic metabolism and adipocyte differentiation. Notably, NK cell-mediated cytotoxicity showed selective enhancement despite comprehensive immune suppression, representing an energy-efficient innate defense strategy. This study provides the first characterization of seasonal immune dynamics in a high-altitude cervid species. These findings reveal persistent immune constraints in high-altitude populations and provide theoretical foundations for disease prevention and health management in captive forest musk deer at high altitudes.

## Simple Summary

This study investigates how captive forest musk deer, a critically endangered species in China, adapt to the challenging conditions of high-altitude environments, including low oxygen, cold temperatures, and intense ultraviolet radiation. By analyzing blood samples from deer living at different altitudes across different seasons, we found that deer at high altitudes suppress their immune systems during autumn-winter to conserve energy, while maintaining basic defense functions through natural killer cells. During spring-summer, their immune system partially recovers, but overall remains weaker than deer at lower altitudes. These findings reveal that high-altitude deer face persistent health challenges due to the trade-off between energy conservation and immune defense. This research is important for the conservation of forest musk deer, as it helps identify vulnerable periods when these animals are more susceptible to diseases. The results can guide disease prevention strategies and improve health management practices in captive breeding facilities, supporting the conservation and sustainable management of this endangered species.

## 1. Introduction

The harsh climatic conditions of high-altitude regions, including hypoxia, low temperatures, intense ultraviolet radiation, and diurnal temperature fluctuations, impose significant ecological pressures on mammals and present complex challenges to animals’ physiological metabolism, energy homeostasis, and immune functions. A decline in oxygen partial pressure not only limits aerobic metabolic efficiency but may also interfere with the energy allocation required for immune responses, potentially triggering widespread transcriptional reprogramming [1,2,3]. The heat loss caused by low temperatures forces animals to increase thermogenesis and insulation mechanisms to maintain body temperature, which consumes a significant amount of energy. This energy expenditure may suppress the function of certain immune cells, reducing pathogen clearance efficiency [4,5,6]. Some studies have found that the incidence of certain diseases increases significantly when animals from lowland areas are introduced to high-altitude regions [7]. This phenomenon suggests that the extreme high-altitude environment may challenge immune defense, making high-altitude adaptation particularly important for mammalian health.

In recent years, with the deepening study of high-altitude immune adaptation mechanisms, more findings indicate that species in high-altitude environments undergo significant immune system remodeling and changes as part of their adaptation to hypoxic conditions [8,9,10]. For example, under hypoxic conditions, the alveolar type II epithelial cells of yaks promote cell proliferation and enhance tolerance to hypoxia by activating the *HIF-1α*/*EGF*/*EGFR* signaling pathway, thereby improving immune response adaptability [11]. Furthermore, the hypoxic environment at high altitudes not only affects metabolic activity but may also inhibit immune cell activity and reduce pathogen response capacity [12]. Therefore, high-altitude animals cope with environmental immune challenges through various physiological mechanisms, including improved blood oxygen-carrying capacity, regulation of immune cell activity and adjusting energy allocation.

Seasonal variation in high-altitude environments further compounds these challenges. Low temperatures, reduced oxygen levels, and food scarcity during autumn-winter may induce energy-conserving immune responses, while the relatively mild climate and increased ultraviolet radiation of spring-summer may trigger distinct metabolic and immune reprogramming. Previous studies have rarely considered seasonal variation as a variable, potentially leading to an incomplete understanding of high-altitude adaptation of mammals. Therefore, comparative studies across different seasons at both high and low altitudes can provide a more comprehensive analysis of the immune-ecological adaptations of plateau species and fill this research gap.

The forest musk deer (*Moschus berezovskii*) is a critically endangered species in China and an important resource animal. The musk secreted by male forest musk deer is used as a fixative in high-end perfumes and as a valuable traditional medicine in various emergency resuscitation and cardiotonic treatments. However, the long-term excessive demand for musk has led to widespread poaching of wild forest musk deer, resulting in a sharp decline in their population and severe damage to wild resources [13]. To protect this endangered species and ensure musk supply, China has begun exploring the artificial breeding of forest musk deer since the 1950s [13,14]. Despite some research progress, large-scale breeding still faces challenges due to genetic issues (including inbreeding depression and loss of genetic diversity) and disease-related issues [15,16]. Forest musk deer are naturally shy and sensitive to disturbances, making them vulnerable to stress and pathogen invasions in captivity. Disease has become one of the major threats to forest musk deer [17]. Therefore, strengthening the research on immune health and improving disease resistance in captive forest musk deer are of great significance.

High-altitude environments impose multiple ecological stressors on captive wildlife, including chronic hypoxia, low ambient temperatures, and elevated ultraviolet radiation, all of which may significantly influence immune function and physiological adaptation. However, there is still a lack of in-depth research on the immune mechanisms of forest musk deer in high-altitude environments [18,19,20]. Therefore, studying the impact of high-altitude environments on the immune function of forest musk deer and its regulatory mechanisms is of great importance for improving health management and disease prevention in captive forest musk deer at high altitudes.

In recent years, transcriptomics technology has been widely applied in high-altitude adaptation research, providing important insights into the molecular basis of immune regulation in high-altitude animals. Through transcriptomic sequencing, researchers are able to comprehensively analyze changes in gene expression and the regulatory networks of the immune system in animals [21,22,23]. This study aimed to reveal the molecular mechanisms of immune adaptation in captive forest musk deer at high altitude through comparative blood transcriptome analysis across two key dimensions: (1) altitudinal differences between high-altitude (~3900 m) and low-altitude (~1450 m) populations, and (2) seasonal dynamics between autumn-winter and spring-summer periods. We focused on immune-related genes and regulatory pathways to characterize year-round adaptive patterns under high-altitude stressors. This study not only fills a gap in high-altitude forest musk deer immunology research but also provides theoretical foundations and practical guidance for disease prevention and health management in this species.

## 2. Material and Methods

### 2.1. Sample Collection

To assess both altitudinal and seasonal effects on immune function, we designed a comparative study with four experimental groups: autumn-winter high-altitude (HA, *n* = 4), autumn-winter low-altitude (LA, *n* = 8), spring-summer HA (*n* = 12), and spring-summer LA (*n* = 9). Three main comparisons were performed: (1) autumn-winter HA vs. LA to evaluate altitude effects under energy-limited conditions; (2) spring-summer HA vs. LA to assess altitude effects under relatively favorable conditions; and (3) spring-summer vs. autumn-winter within the HA group to examine seasonal immune dynamics at high altitude.

**Figure 1:**
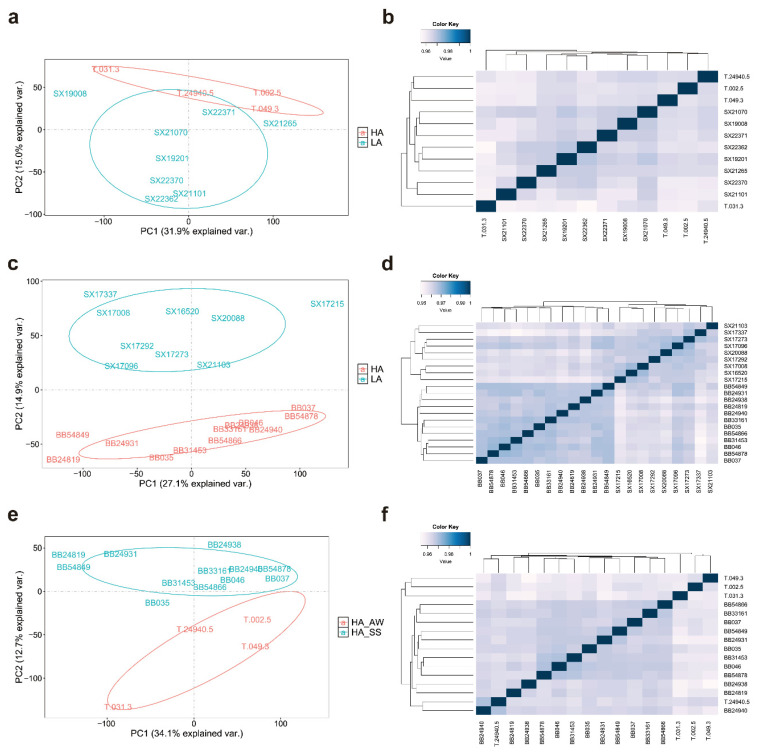
Principal component analysis and hierarchical clustering of transcriptome samples across altitude and seasonal comparisons. (**a**,**b**) Autumn-winter comparison between high-altitude and low-altitude groups; (**c**,**d**) Spring-summer comparison between high-altitude and low-altitude groups; (**e**,**f**) Seasonal comparison within the high-altitude group. (**a**,**c**,**e**) Principal component analysis (PCA) plots showing sample distribution along the first two principal components (PC1 and PC2), with percentage of variance explained indicated on each axis; (**b**,**d**,**f**) Hierarchical clustering heatmaps based on Spearman correlation coefficients between samples. HA: high altitude (~3900 m); LA: low altitude (~1450 m); AW: autumn-winter; SS: spring-summer.

A total of 33 healthy adult forest musk deer were selected as study subjects (Table 1). All animals were aged 3–6 years with no recent history of disease or drug treatment. While age and sex were recorded for all individuals, the study focused on altitude and seasonal effects as primary biological variables. All animals were mature adults within this narrow age range, which is characterized by stable immune function in mammals [24,25]. Unsupervised clustering analyses (PCA and hierarchical clustering, Figure 1) confirmed that transcriptomic variation was predominantly driven by altitude and season rather than age or sex, validating our focus on these primary variables.

Twelve autumn-winter samples included 4 from captive forest musk deer at the high-altitude site (Bianba County, Chamdo City, Tibet Autonomous Region; ~3900 m elevation; High-Altitude Group, HA) and 8 from captive forest musk deer at the low-altitude site (Feng County, Shanxi Province; ~1450 m elevation; Low-Altitude Group, LA). The high-altitude facility represents the highest musk deer breeding base in China, characterized by hypoxic conditions, low ambient temperatures, and intense ultraviolet radiation. In contrast, the low-altitude site is located in a warm temperate zone with a warm and humid climate. Twenty-one spring-summer samples included 12 captive forest musk deer from the same high-altitude region (HA group) and 9 from the low-altitude region (LA group). Sample collection at high altitude was constrained by several practical factors: limited captive population at the Bianba County facility (~3900 m), harsh autumn-winter sampling conditions (extreme cold, heightened deer stress sensitivity), and stringent inclusion criteria (age 3–6 years, healthy status, outside breeding season). These constraints resulted in a relatively small sample size for the autumn-winter high-altitude group (*n* = 4). However, stringent quality control and appropriate statistical methods (detailed in Section 2.6) were implemented to ensure reliability despite this limitation.

The breeding season of captive forest musk deer extends from late October to February [26,27]. All sampling was conducted outside this period: autumn-winter samples were collected in mid-October, and spring-summer samples were collected in late June. This timing minimized the confounding effects of reproductive hormones on immune and metabolic gene expression.

Blood collection was performed without anesthesia using a standardized physical restraint protocol. Deer were acclimated in a small holding pen 1–2 h prior to collection. An experienced veterinarian restrained the deer by grasping the hind legs, lifting the lower legs below the joint, rotating the deer to the left side, and securing the forelimbs below the carpal joint. The deer was positioned horizontally across the veterinarian’s lap, avoiding pressure on the heart and rumen. An assistant held the deer’s head and covered the eyes with a cloth (ensuring unobstructed nasal passages) to calm the animal.

Approximately 2.5 mL of blood was collected via sterile venipuncture from the forelimb vein using BD PAXgene Whole Blood RNA Collection Tubes (BD Biosciences, Franklin Lakes, NJ, USA), which contain a proprietary RNA stabilization reagent that immediately inhibits RNA degradation and prevents ex vivo gene expression changes upon blood collection. After collection, tubes were inverted 8–10 times to ensure thorough mixing with the stabilization reagent. Samples were immediately stored at −20 °C or −80 °C for long-term preservation and transported under cold chain conditions (dry ice) until RNA extraction. All procedures were performed by trained veterinary personnel.

### 2.2. Total RNA Extraction

All experimental procedures and animal care practices followed the guidelines approved by the Experimental Animal Management and Use Committee of Sichuan University (License Number SCU240301019).

Total RNA was extracted from blood samples of the forest musk deer using Trizol reagent (Invitrogen, Carlsbad, CA, USA) according to the manufacturer’s instructions. RNA integrity was assessed using the Agilent 2100 system (2100 Expert Software, version B.02.10), and samples with RNA Integrity Numbers (RINs) greater than 5.8 were selected for RNA-seq library preparation. Total RNA quantification and characterization included: (1) NanoDrop assay for RNA purity and concentration; (2) Agilent 2100 measurement for RNA concentration and integrity. Only samples meeting requirements proceeded to library construction.

### 2.3. RNA Library Preparation and Sequencing

According to the kit manufacturer’s protocol, mRNA was enriched from total RNA using Oligo (dT) magnetic beads. Fragmentation buffer was then added to cleave mRNA into short fragments. Using mRNA as a template, the first strand of cDNA was synthesized with a six-base random primer. Subsequently, buffer, dNTPs, DNA polymerase I, and RNase H were added to synthesize the second strand of cDNA. Double-stranded cDNA was purified using AMPure XP beads. Following purification, end repair, A-tailing, and sequencing adapter ligation were performed. Fragment size selection was achieved using AMPure XP beads. Finally, PCR amplification was conducted, and the product was purified with AMPure XP beads to obtain the library. Following library construction, initial quantification was performed using Qubit 2.0, followed by library dilution. Insert size was assessed via Agilent 2100 analysis. Upon confirming expected parameters, precise quantification of effective concentration was achieved through Q-PCR to ensure library quality. Sequencing was conducted on the Illumina NovaSeq Xplus platform.

### 2.4. Raw Sequencing Data Quality Control and Gene Expression Quantification

A comprehensive overview of the bioinformatics analysis workflow is provided in Table S4. Using NGSQC Toolkit (version 2.3.3) to remove adapter sequences and low-quality reads with a quality score below 20 from raw sequencing data [28], followed by FastQC (version 0.11.9) (http://www.bioinformatics.babraham.ac.uk/projects/fastqc/, accessed on 30 December 2024) to assess the quality of filtered data. Reads from each sample were aligned to the forest musk deer reference genome using HISAT2 (version 2.1.0) [29]. The forest musk deer genome was downloaded from GIGADB DATASETS (https://gigadb.org/, accessed on 30 December 2024), DOI: 10.5524/102631 [30]. SAM files were converted to BAM files using Samtools (version 1.9) [31]. Finally, gene expression abundance was estimated using featureCounts from the Subread software package (version 1.6.4) based on the reference genome annotation file [32].

### 2.5. Principal Component Analysis and Spearman Correlation Distance Clustering

To validate the overall expression patterns and intragroup differences among forest musk deer samples across different altitudes and seasons, Principal Component Analysis (PCA) and Spearman correlation distance clustering were employed for evaluation. The same methods were applied to samples from the autumn-winter high-altitude group (HA) and low-altitude group (LA), spring-summer HA and LA, and spring-summer versus autumn-winter HA. Raw gene expression counts were first normalized using the Trimmed Mean of M-values (TMM) method implemented in the edgeR package (version 3.42.4) [33]. The TMM-normalized expression matrix then underwent log2 transformation to stabilize variance and approximate normal distribution. R plots were generated with the ggplot function from the ggplot2 package (version 3.4.4) [34].

For hierarchical clustering analysis, the Spearman correlation coefficient between samples was calculated from the log2-transformed expression matrix using the cor function from the stats package (version 4.3.0) to construct a distance matrix. A heatmap with hierarchical clustering was generated using the heatmap.2 function from the gplots package.

### 2.6. Differential Gene Expression Analysis

To identify differentially expressed genes (DEGs), the edgeR package in R was employed for differential expression analysis [33]. The edgeR package was specifically selected for this study due to its superior performance with modest sample sizes, particularly relevant given our autumn-winter high-altitude group (*n* = 4). EdgeR employs empirical Bayes methods to share information across genes through dispersion estimation, thereby improving statistical power even when biological replicates are limited [35,36]. The same methodology was applied to compare autumn-winter HA vs. LA, spring-summer HA vs. LA, and spring-summer vs. autumn-winter.

The differential expression analysis workflow consisted of the following steps. Low-expression genes were first filtered from the gene expression matrix using the filterByExpr function in edgeR. This function retains genes with a minimum read count of 10 in the smallest sample group, thereby reducing noise from genes with insufficient expression levels for reliable statistical inference. To account for technical variation, sequencing depth, and gene length differences across samples, the filtered expression matrix was normalized using the Trimmed Mean of M-values (TMM) method implemented in edgeR. Based on the TMM-normalized gene expression matrix, differential expression analysis was performed between comparison groups. Genes were considered significantly differentially expressed when meeting both criteria: Benjamini-Hochberg adjusted false discovery rate (FDR) ≤ 0.05 and absolute log2 fold change (|log2FC|) ≥ 1. These dual thresholds balance statistical rigor and biological relevance: FDR ≤ 0.05 controls false discovery rate below 5% across multiple testing, ensuring statistical reliability; |log2FC| ≥ 1 (corresponding to ≥2-fold expression change) identifies biologically meaningful alterations [37,38,39].

### 2.7. Enrichment Analysis

To further explore the biological functions and pathways involved in differentially expressed genes (DEGs), this study performed Gene Ontology (GO) functional enrichment analysis and Kyoto Encyclopedia of Genes and Genomes (KEGG) pathway enrichment analysis on significantly differentially expressed genes, using the forest musk deer genome as the background gene set.

First, GO functional annotation information for genes in the forest musk deer background gene set was obtained from the EggNOG v5.0 database (http://eggnog5.embl.de/#/app/home, accessed on 30 May 2025). Concurrently, KEGG pathway annotation information for each gene was extracted from the KEGG PATHWAY database (https://www.kegg.jp/kegg/pathway.html, accessed on 30 May 2025) to construct a comprehensive background gene annotation set. Subsequently, the enricher function in the clusterProfiler (version 4.8.3) package of R was employed to perform enrichment analysis on the DEGs based on the constructed background gene annotation set, identifying significantly enriched functional categories [40]. GO terms or KEGG pathways with a pvalue cutoff of less than 0.05 were considered significantly enriched.

### 2.8. Protein Interaction Network Analysis

To further explore potential functional relationships among differentially expressed genes (DEGs) and identify core genes, this study performed protein-protein interaction (PPI) analysis on significantly differentially expressed DEGs using the STRING database (https://string-db.org, version 12.0) [41]. Network analysis and hub gene screening were conducted using Cytoscape software (version 3.7.1). The same methodology was applied to DEGs between autumn-winter HA and LA, spring-summer HA and LA, and spring-summer vs. autumn-winter in the high-altitude group.

Protein interaction data obtained from the STRING database were filtered using default parameters (interaction score > 0.4), and interaction relationship files were exported. Cytoscape software (version 3.7.1) was used to construct PPI network diagrams for DEGs [42]. Further network analysis tools in Cytoscape were employed to calculate the connectivity (degree) of each gene node. Genes with higher connectivity were defined as hub genes within the network.

## 3. Results

### 3.1. Overview of RNA-Seq Data

This study obtained RNA sequencing data from 33 peripheral blood samples of the forest musk deer, including 12 autumn-winter samples (4 from the high-altitude group (HA) and 8 from the low-altitude group (LA)) and 21 spring-summer samples (12 from HA and 9 from LA) (see Table 2). Following paired-end 150 bp sequencing on the Illumina NovaSeq Xplus platform, autumn-winter samples yielded an average of 117,998,268 raw reads, with an average of 115,885,773 clean reads, representing a clean read rate of 98.62%. The average Q20 and Q30 values were 97.78% and 94.05%, respectively. The average GC content was 52.76%. The mapping rate ranged from 95.80% to 97.76%, with an average of 96.67%. For spring-summer samples, the average raw read count was 49,894,823, with an average clean read count of 49,143,859 and a cleaning rate of 98.45%. The average Q20 and Q30 values were 97.55% and 93.69%, respectively. The average GC content was 50.15%. The mapping rate ranged from 96.72% to 98.46%, with an average of 97.93%.

### 3.2. Principal Component Analysis (PCA) and Spearman Correlation Distance Clustering

This study performed principal component analysis and Spearman correlation distance hierarchical clustering on the TMM-normalized gene expression counts of all samples.

For autumn-winter samples (Figure 1a,b), PCA revealed clear separation between HA and LA groups, with PC1 and PC2 collectively explaining 46.9% of total variance. HA samples clustered distinctly along the PC2 axis, while LA samples grouped separately with mild dispersion. Spearman correlation-based hierarchical clustering confirmed this altitude-driven grouping pattern.

For spring-summer samples (Figure 1c,d), PCA similarly showed distinct altitude-based clustering (PC1 + PC2: 42.0% variance). HA samples clustered tightly while LA samples exhibited mild dispersion. Hierarchical clustering confirmed elevation as the primary driver of transcriptomic variation.

For the seasonal comparison within the HA group (Figure 1e,f), PCA revealed clear separation between spring-summer and autumn-winter samples (PC1 + PC2: 46.8% variance). Hierarchical clustering confirmed distinct seasonal grouping, indicating significant seasonal transcriptomic changes in high-altitude forest musk deer.

### 3.3. Analysis of Differentially Expressed Genes

**Figure 2:**
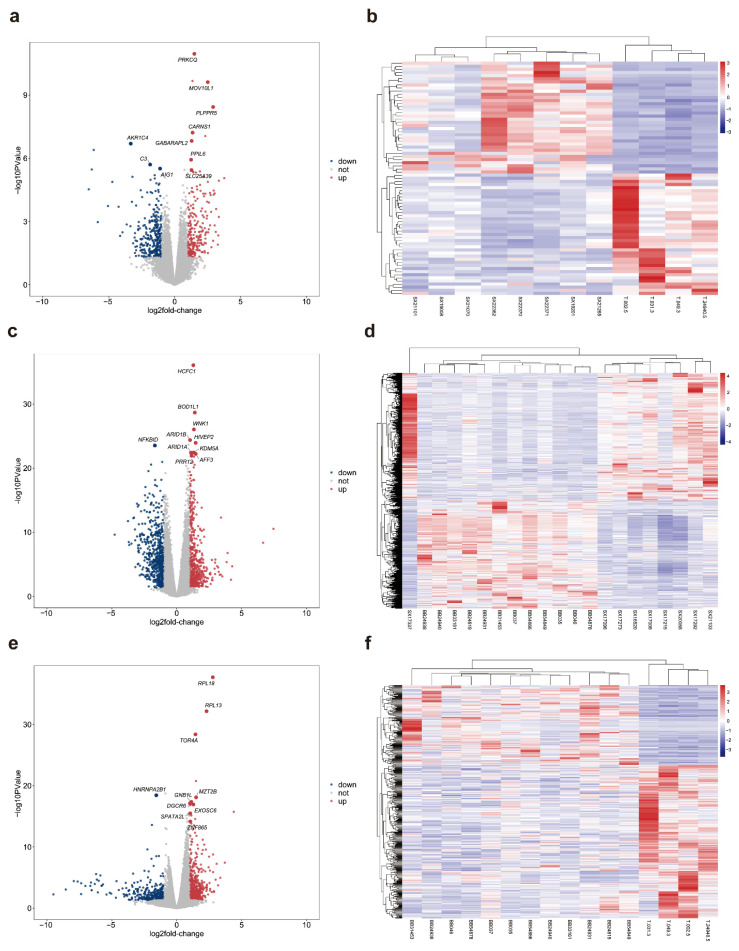
Differential gene expression patterns revealed by volcano plots and heatmaps across altitude and seasonal comparisons. (**a**,**b**) Autumn-winter comparison between high-altitude and low-altitude groups; (**c**,**d**) Spring-summer comparison between high-altitude and low-altitude groups; (**e**,**f**) Seasonal comparison (spring-summer vs. autumn-winter) within the high-altitude group. (**a**,**c**,**e**) Volcano plots displaying differentially expressed genes (DEGs), with log2 fold change (log2FC) on the *x*-axis and −log10 PValue on the *y*-axis. Red dots represent significantly upregulated genes, blue dots represent significantly downregulated genes, and gray dots represent non-significant genes. The top 10 DEGs ranked by FDR are highlighted with larger points and labeled with gene names in italics. (**b**,**d**,**f**) Heatmaps showing hierarchical clustering of DEGs across samples, with color scale representing normalized gene expression levels (red: high expression, blue: low expression).

For autumn-winter HA vs. LA, a total of 82 significantly differentially expressed genes were identified, including 46 upregulated genes and 36 downregulated genes (HA relative to LA). Volcano plot results (Figure 2a) indicate that most DEGs fall within the ±3 range of log_2_(FC). Heatmap clustering analysis based on DEG expression profiles (Figure 2b) reveals a clear expression boundary between the HA and LA groups. The top ten differentially expressed genes ranked by FDR significance are listed in Table S1, comprising 3 upregulated genes and 7 downregulated genes. These genes primarily involve immune-related (*FAM26F*, *IFIT3*, *PRKCQ*, *AIRE*), metabolism-related (*LIPA*), cell structure-related (*MYOF*, *EPB42*), and neuro-related (*CHRM5*) pathways.

For spring-summer HA vs. LA, the analysis identified a total of 907 significantly differentially expressed genes, comprising 426 upregulated genes and 481 downregulated genes (HA relative to LA). Volcano plot results (Figure 2c) indicate that most DEGs fall within the ±3 range of log_2_(FC). Heatmap clustering analysis based on DEG expression profiles (Figure 2d) reveals a clear expression boundary between the HA and LA groups. The top ten differentially expressed genes ranked by FDR significance are listed in Table S2, comprising 8 upregulated genes and 2 downregulated genes. These genes primarily involved immune-related (*MB21D1*), metabolic-related (*PHIP*, *FOXO3*, *SMPDL3A*), DNA repair-related (*REV3L*), cell structure and communication-related (*COL12A1*, *MYO6*, *GJA1*), and stress-related (*LCA5*) pathways.

For the high-altitude group comparing spring-summer versus autumn-winter, a total of 425 significantly differentially expressed genes were identified, comprising 281 upregulated genes and 144 downregulated genes (spring-summer relative to autumn-winter). Volcano plot results (Figure 2e) indicate that most DEGs fall within the ±4 range of log_2_(FC). Heatmap clustering analysis based on DEG expression profiles (Figure 2f) reveals clear expression boundaries between spring-summer and autumn-winter groups. The top ten differentially expressed genes ranked by FDR significance are listed in Table S3, including 4 upregulated genes and 6 downregulated genes. These genes primarily involve immune-related (*HRH2*, *PTGER2*), neuro-related (*HTR1B*), cell structure-related (*GJA1*, *TMSB10*), RNA/protein processing-related (*HNRNPA2B1*, *RPL13*), and ion channel-related (*POPDC3*) functions.

### 3.4. Go and Kegg Functional Enrichment Analysis

To reveal changes in immune and metabolic functions among groups, we performed GO and KEGG enrichment analyses on DEGs, focusing on immune-related pathways (innate and adaptive immunity) and metabolic pathways (carbohydrate, lipid, protein, and amino acid metabolism).

**Figure 3:**
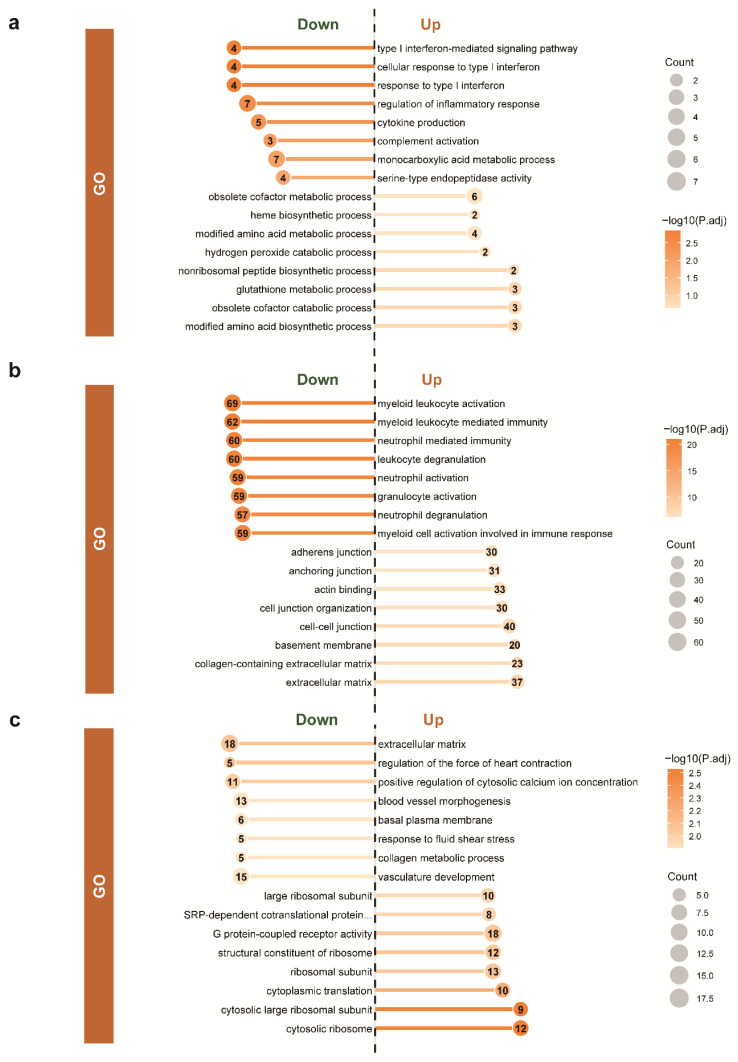
Gene Ontology (GO) enrichment analysis of differentially expressed genes across altitude and seasonal comparisons. (**a**) Autumn-winter comparison between high-altitude (HA) and low-altitude (LA) groups; (**b**) Spring-summer comparison between HA and LA groups; (**c**) Seasonal comparison (spring-summer vs. autumn-winter) within the HA group. The top 8 most enriched GO terms are shown for both upregulated (right, positive direction) and downregulated (left, negative direction) genes. Bar length represents enrichment significance (−log10 adjusted *p* value). Circle size indicates the number of genes (Count) involved in each GO term, and circle color reflects the −log10 adjusted *p* value (darker color indicates higher significance).

**Figure 4:**
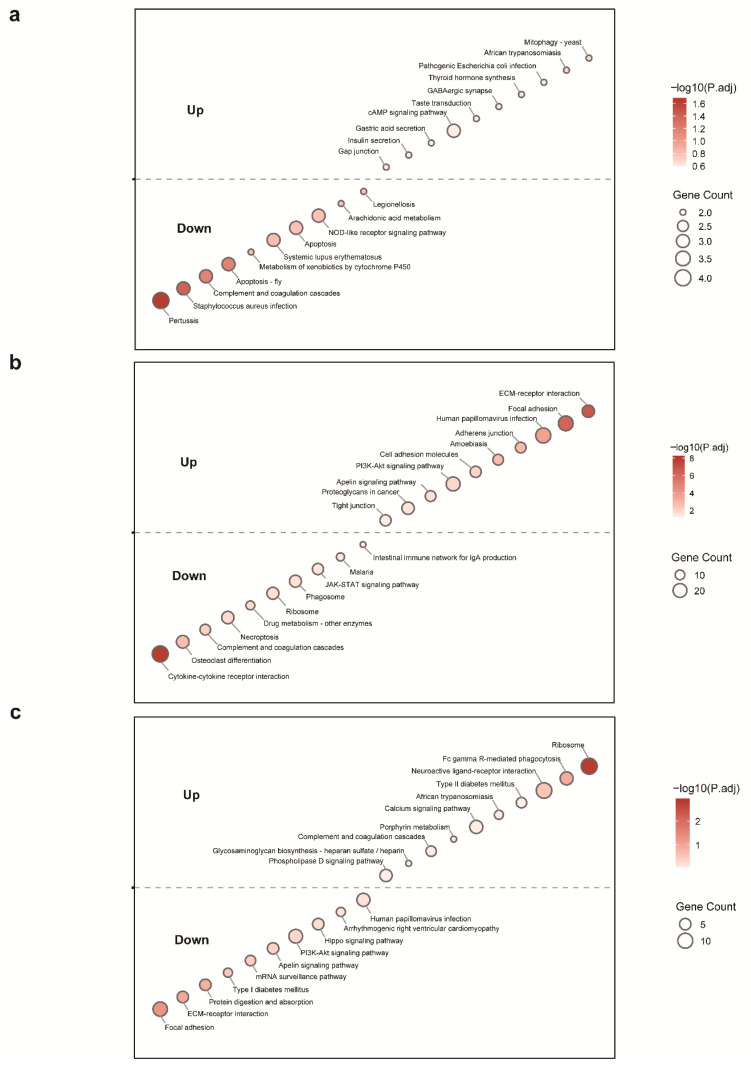
Kyoto Encyclopedia of Genes and Genomes (KEGG) pathway enrichment analysis of differentially expressed genes across altitude and seasonal comparisons. (**a**) Autumn-winter comparison between high-altitude (HA) and low-altitude (LA) groups; (**b**) Spring-summer comparison between HA and LA groups; (**c**) Seasonal comparison (spring-summer vs. autumn-winter) within the HA group. The top 10 most enriched KEGG pathways are shown for both upregulated and downregulated genes. Bubbles above and below the dashed line correspond to pathways enriched in upregulated and downregulated genes, respectively. Bubble size indicates the number of genes (Gene Count) involved in each pathway, and bubble color represents the −log10 adjusted *p* value (darker color indicates higher significance).

For autumn-winter HA vs. LA (Figure 3a and Figure 4a), the HA group exhibited comprehensive immune suppression. Innate immunity showed significant downregulation of complement system pathways (complement activation, complement and coagulation cascades) and interferon regulation (type I/II interferon production). Adaptive immunity was similarly constrained, with downregulation of T cell-related pathways (T cell activation/proliferation, Th1 response) and humoral immune responses (B cell-mediated immunity). Notably, natural killer cell-mediated cytotoxicity showed selective upregulation despite overall immune suppression. Metabolically, the HA group displayed adaptive reprogramming characterized by: (1) enhanced anaerobic glycolysis (upregulated lactate/pyruvate metabolism); (2) suppressed lipid biosynthesis (fatty acid, cholesterol, triglyceride synthesis) with concurrent fat mobilization (lipase activity); and (3) reduced protein degradation (peptidase activity) with upregulated amino acid metabolism (histidine, alanine-aspartate-glutamate pathways). These patterns reflect an energy-conserving metabolic mode during autumn-winter.

For spring-summer HA vs. LA (Figure 3b and Figure 4b), immune pathways remained predominantly downregulated compared to LA, though partial recovery was evident. Innate immunity showed persistent suppression of neutrophil-mediated immunity, interferon responses, and complement regulation, but phagocytosis-recognition and NK cell cytotoxicity were upregulated. Adaptive immunity exhibited continued T cell suppression (T cell activation/proliferation, Th1 responses) but enhanced humoral components including lymphocyte activation, B cell differentiation, and NF-κB signaling. Metabolically, the spring-summer HA group showed a shift toward anabolism: (1) upregulated glucose homeostasis and carbohydrate metabolism; (2) enhanced adipocyte differentiation (both white and brown) with suppressed lipolysis; and (3) increased protein synthesis with reduced proteolysis. These patterns indicate metabolic optimization toward energy storage during the relatively favorable spring-summer period.

For spring-summer vs. autumn-winter within the HA group (Figure 3c and Figure 4c), significant immune enhancement was observed. Innate immunity showed upregulation of neutrophil-mediated immunity, antibacterial responses, and myeloid cell activation. Adaptive immunity exhibited enhanced Th cell activation (CD4+ αβ T cell pathways), Fcγ receptor-mediated phagocytosis, and B cell activation/proliferation pathways. Metabolically, lipid synthesis (adipocyte differentiation) and protein biosynthesis were upregulated, while protein degradation pathways were suppressed. These changes indicate significantly enhanced metabolic activity during spring-summer compared to autumn-winter.

Seasonal comparisons reveal that immune activity in autumn-winter is predominantly suppressed, supporting energy conservation. In spring-summer, although immune activity remains relatively low compared to low-altitude forest musk deer, it is significantly upregulated relative to autumn-winter, indicating heightened metabolic activity and vigorous life processes.

### 3.5. PPI Network Construction and Core Gene Identification

To further explore potential functional relationships among differentially expressed genes (DEGs) and identify core genes playing key roles in the high-altitude adaptation of the forest musk deer, this study performed protein-protein interaction (PPI) analysis on significantly differentially expressed DEGs using the STRING database. Network analysis and core gene (hub gene) screening were conducted using Cytoscape software.

**Figure 5:**
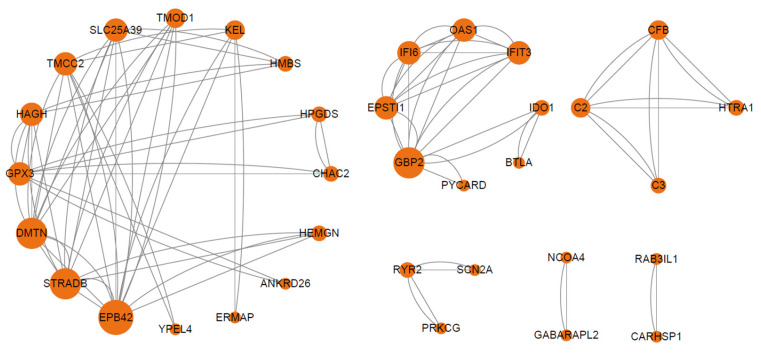
Protein-protein interaction (PPI) network of differentially expressed genes in autumn-winter comparison between high-altitude and low-altitude groups. The sub-network contained hub genes was extracted. Nodes represent proteins encoded by DEGs, with node size proportional to the degree of connectivity (number of interactions).

For autumn-winter HA vs. LA (Figure 5), the PPI network comprised 35 nodes and 96 edges (average degree: 5.49). Six modules were identified. Among them, immune-related modules included Module 2 (Immune Regulation, core genes *GBP2*, *EPSTI1*, *IFI6*, *OAS1*, *IFIT3* downregulated; *BTLA* upregulated) and Module 3 (Complement System Inhibition, core genes *C2*, *C3*, *CFB* downregulated). Metabolism-related modules included Module 1 (Red Blood Cell Structure and Oxidative Stress Response, core genes *EPB42*, *STRADB*, *DMTN*, *GPX3*, *HAGH*, *SLC25A39* upregulated) and Module 5 (iron metabolism and autophagy regulation, core genes *GABARAPL2*, *NCOA4* upregulated). These modules indicate that immune interactions are predominantly inhibitory, while metabolic interactions enhance oxidative stress tolerance and iron homeostasis.

**Figure 6:**
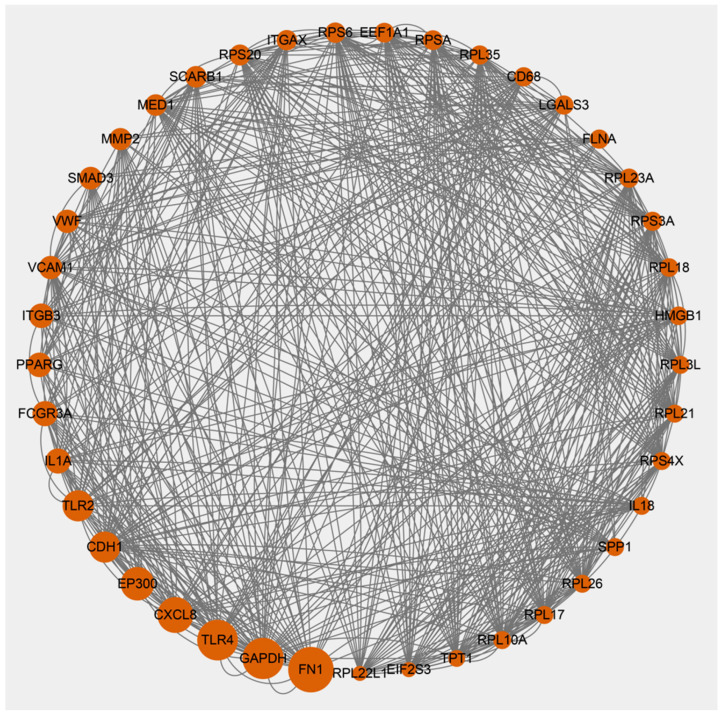
Protein-protein interaction (PPI) network of differentially expressed genes in spring-summer comparison between high-altitude and low-altitude groups. The sub-network contained hub genes was extracted. Nodes represent proteins encoded by DEGs, with node size proportional to the degree of connectivity (number of interactions).

For spring-summer HA vs. LA (Figure 6), the PPI network comprised 545 nodes and 3700 edges (average degree: 13.57). Top hub genes included *FN1* (upregulated; extracellular matrix), *GAPDH* (downregulated; glycolysis), *TLR4*/*TLR2* (downregulated; innate immune recognition), and *CXCL8* (downregulated; inflammation), reflecting suppressed inflammatory signaling with enhanced tissue remodeling.

**Figure 7:**
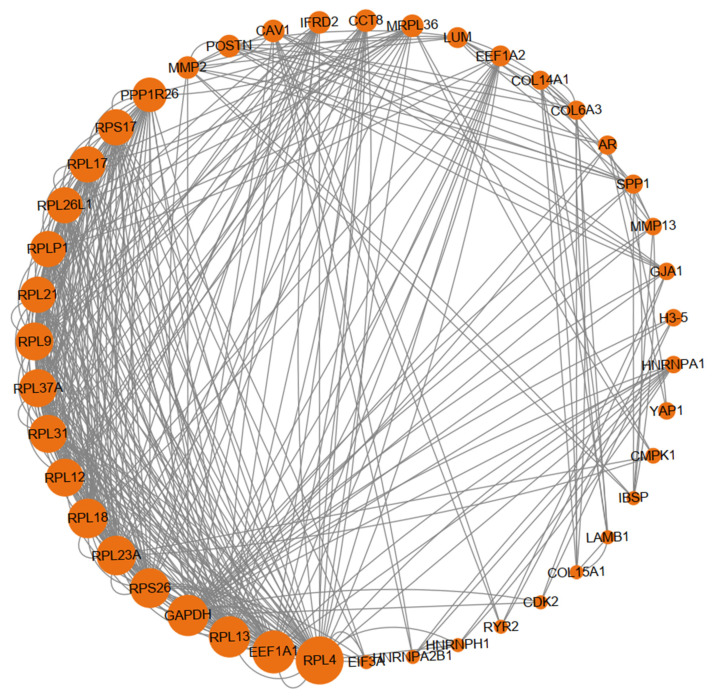
Protein-protein interaction (PPI) network of differentially expressed genes in seasonal comparison within the high-altitude group. The sub-network contained hub genes was extracted. Nodes represent proteins encoded by DEGs, with node size proportional to the degree of connectivity (number of interactions).

For spring-summer vs. autumn-winter within the HA group (Figure 7), the PPI network comprised 403 nodes and 306 edges (average degree: 1.52). Hub genes were predominantly ribosomal proteins (RPL4, RPL13, RPL18, RPS26—upregulated) and translation factors (EEF1A1—upregulated), indicating enhanced protein synthesis capacity consistent with the metabolic shift toward anabolism during spring-summer.

## 4. Discussion

### 4.1. Adaptive Characteristics of the Immune System in High-Altitude Forest Musk Deer

High-altitude environments induce immune system remodeling to balance energy allocation and pathogen defense demands [12,43]. Through comparative blood transcriptomic analysis, this study reveals adaptive patterns in the immune dynamics of captive forest musk deer during autumn-winter and spring-summer seasons. Results indicate that the forest musk deer immune system does not follow a simple seasonal suppression-recovery cycle but instead exhibits a refined functional restructuring strategy: shifting from comprehensive energy conservation in autumn-winter to selective enhancement of innate and adaptive immunity in spring-summer.

Previous genomic and transcriptomic studies on high-altitude mammals have primarily focused on hypoxia adaptation mechanisms. The landmark yak (*Bos grunniens*) genome study revealed expansion of gene families related to sensory perception and energy metabolism, with positively selected genes enriched in hypoxia and nutrition metabolism pathways [44]. Similarly, the Tibetan antelope (*Pantholops hodgsonii*) genome showed adaptive evolution in genes associated with energy metabolism and oxygen transmission, with convergent evolution in hypoxia-related genes [45]. Comparative transcriptome analyses between yak and cattle further demonstrated enhanced activation of immune-related pathways in high-altitude adapted bovids [46]. However, these studies have primarily characterized genetic adaptations for hypoxia tolerance rather than the dynamic immune-metabolic trade-offs that may occur seasonally. Our study addresses this gap by providing the first systematic characterization of seasonal immune dynamics in a high-altitude cervid species, revealing not only the expected patterns of immune suppression but also several unexpected adaptive mechanisms.

#### 4.1.1. Immunological Characteristics in Autumn-Winter

Transcriptome comparison between the HA and LA during autumn-winter revealed large-scale suppression of the immune function in HA. Innate immune pathways were broadly downregulated, with the complement system significantly inhibited (downregulation of *CFB*/*C2*/*C3*). These components are essential for complement activation across alternative (*CFB*), classical, and lectin pathways (*C2*), with *C3* serving as the central hub molecule [47]. Their coordinated downregulation impairs complement-mediated pathogen clearance and inflammatory amplification, reducing rapid immune response capabilities but conserving substantial metabolic resources. This trade-off has direct implications for captive management: reduced complement function during autumn-winter increases susceptibility to bacterial infections, suggesting that enhanced biosecurity and environmental hygiene are particularly critical during this period. Suppression of interferon pathways (*FLT3* downregulation) further restricts antiviral defense by limiting dendritic cell development and type I interferon production [48]. This reduced antiviral capacity suggests that viral disease outbreaks may pose heightened risks during autumn-winter, warranting vigilant health monitoring. Downregulation of these innate immune pathways and genes indicates that high-altitude forest musk deer reduce rapid immune responses during autumn-winter, diminishing non-specific inflammatory cascades and antiviral capabilities to conserve energy in cold environments. Suppression of adaptive immunity is more pronounced compared to low-altitude groups. Regarding cellular immunity, T cell proliferation and activation pathways are significantly downregulated (*PYCARD*/*EBI3*). Downregulation of *PYCARD* may limit T cell activation and proliferation, contributing to reduced adaptive immune responses. *EBI3* is a subunit of IL-27, which promotes T cell proliferation and Th1 differentiation [49]. Downregulation of *EBI3* may impair IL-27-mediated T cell activation. Humoral immunity is similarly constrained, with downregulation of genes associated with B cell-mediated immune responses (*CFB*/*C2*/*C3*). The complement component C3 cleavage product C3d binds to CR2 to form a B cell co-receptor complex. This complex amplifies BCR signaling and is essential for antibody responses to T cell-dependent antigens. Weakened C3d-CR2 signaling directly inhibits B cell activation, proliferation, and germinal center responses, limiting high-affinity antibody production and class switching [50]. Protein-protein interaction (PPI) network analysis further confirms immune suppression in high-altitude forest musk deer. Interferon-stimulated genes (ISGs) in the immune module, such as *IFIT3*, are significantly downregulated. *IFIT3* inhibits translation initiation by recognizing viral RNA lacking 2′-O-methylation, thereby weakening antiviral defense. Downregulation of complement component C3, a network hub node, triggered a cascade of inhibitory effects, impacting multiple downstream functions including complement activation, B-cell co-stimulation, and opsonization.

However, against this backdrop of widespread inhibition, NK cell-mediated cytotoxicity exhibits selective maintenance (PRKCQ upregulation). NK cells possess a unique energetic advantage: they rapidly eliminate infected and stressed cells without requiring antigen presentation or clonal expansion, representing the most energy-efficient cytotoxic mechanism available under resource constraints [51]. This selective preservation of NK cell function while suppressing energy-intensive adaptive responses exemplifies the selective immune enhancement strategy employed by high-altitude forest musk deer year-round, as confirmed by consistently elevated NK cell activity compared to low-altitude populations across both seasons.

Downregulation of innate immunity during autumn-winter (e.g., reduced complement cascade activation) lowers infection surveillance efficiency, promoting energy conservation but potentially weakening immediate pathogen clearance. Downregulation of adaptive immunity (e.g., restricted T/B cell responses) may weaken long-term defense and increase susceptibility to bacterial infections, such as pyogenic pathogens. However, NK cell upregulation helps maintain fundamental immune protection. This selective immune regulation strategy holds significant ecological adaptive significance, rooted in the energy constraints of high-altitude environments during autumn-winter. The dual stressors of hypoxia and cold in high-altitude environments during autumn-winter cause severe energy shortages: reduced atmospheric oxygen partial pressure limits aerobic metabolic efficiency, while low temperatures increase demands for thermoregulation and heat production. Against this backdrop, the immune system—a highly energy-intensive physiological activity (T-cell clonal expansion requires substantial ATP and biosynthetic precursors, while antibody-producing plasma cells rank among the most metabolically active cells)—becomes a primary target for energy conservation. Inhibition of the complement cascade prevents excessive inflammation and tissue damage caused by cascade amplification, particularly critical in hypoxic conditions. Suppression of adaptive immunity conserves substantial energy normally expended on lymphocyte proliferation, antibody synthesis, and immune memory maintenance, redirecting it toward survival-essential processes like thermoregulation, basal metabolism, and vital organ function. However, this immunosuppression carries potential health risks. The extensive downregulation of innate immunity (complement, interferons, inflammasomes) markedly reduces early pathogen recognition and rapid clearance capabilities, potentially increasing susceptibility to bacteremia and viral infections. Suppression of adaptive immunity limits the establishment of specific immune memory and the production of high-affinity antibodies, proving particularly disadvantageous for chronic infections requiring long-term immune protection. Thus, immune suppression during autumn-winter represents a trade-off of “sacrificing defensive completeness to ensure survival priority”. Under artificial captive conditions, navigating this immunologically vulnerable period requires reducing pathogen exposure (enhancing environmental hygiene and biosecurity), optimizing nutritional support to mitigate energy constraints, and carefully evaluating the timing of immune challenges such as vaccinations based on individual health status and disease risk.

#### 4.1.2. Immunological Characteristics in Spring-Summer

During spring-summer, innate immunity shows significant enhancement. Neutrophil-mediated immune pathways (*SLPI*, *NCF1*, *ELANE*) are upregulated, restoring core antimicrobial functions including phagocytosis, respiratory burst, and NET formation [52,53,54,55,56,57]. This recovery is crucial for countering increased bacterial loads in warmer spring-summer conditions. However, neutrophil activity remains lower than in low-altitude populations, indicating that high-altitude deer retain elevated susceptibility to bacterial infections even during favorable seasons. Nutritional supplementation to support neutrophil production may be beneficial. Enhanced innate antibacterial responses (upregulation of *ELANE*) further support antimicrobial defense. Neutrophil elastase directly degrades bacterial outer membrane proteins and virulence factors [56,57]. However, despite significant recovery from autumn-winter to spring-summer, neutrophil-mediated immunity in the spring-summer high-altitude group remained markedly weaker than that in the low-altitude group during the same season. This indicates that even with some environmental improvement, long-term exposure to high altitude imposes constraints on basal metabolic rate, oxygen delivery capacity, and energy reserves, resulting in persistently lower immunity compared to low-altitude forest musk deer. Neutrophils are continuously produced at high rates in the bone marrow—a process demanding substantial energy expenditure [58]. In energy-limited high-altitude environments, the metabolic cost of sustaining neutrophil production and function imposes constraints on adaptive capacity. Beyond neutrophil functional recovery, the spring-summer HA group also exhibited enhanced Type I interferon responses. Although gene expression in pathways related to interferon-α and interferon-β responses remained lower than in the LA group (downregulation of ISGs such as *OAS1* and *IFITM3*), compared to the autumn-winter high-altitude group, the type I interferon production pathway was significantly upregulated. Specifically, *MB21D1* (cGAS), which recognizes cytoplasmic DNA to initiate the interferon response, along with transcriptional regulators *ZBTB16* and *ZBTB20*, showed significant upregulation. This enhanced cGAS-STING pathway activation helps counteract DNA viruses or intracellular bacteria during spring-summer [59]. Nevertheless, downstream interferon-stimulated gene (*ISG*) expression remained lower than in the low-altitude group, indicating that interferon signaling amplification and antiviral state establishment remain partially constrained.

NK cell function showed further enhancement during spring-summer, with *PRF1* (perforin) upregulation indicating increased cytotoxic capacity [60]. This enhancement compensates for persistent T cell dysfunction, as NK cells can partially substitute for constrained CD8+ CTL responses in eliminating intracellular pathogen-infected cells. From a management perspective, the robust NK cell function suggests that high-altitude deer retain effective surveillance against virus-infected and stressed cells, though this should not be relied upon as a substitute for comprehensive disease prevention programs.

The white blood cell transendothelial migration pathway showed upregulation in the spring-summer high-altitude group compared to the low-altitude group (including *VCL*, *CDH5*, *MMP2*, *ITGAX*, and *VCAM1*), indicating enhanced expression of endothelial adhesion molecules and extracellular matrix remodeling enzymes. This upregulation suggests an increased capacity for leukocyte recruitment from circulation to tissues. However, the functional significance of this enhanced recruitment pathway remains constrained by the limited availability and functionality of circulating myeloid cells. This pattern may reflect a compensatory attempt to maximize immune cell deployment to sites of infection or injury, though the effectiveness of this mechanism is likely limited by the reduced numbers and impaired function of circulating leukocytes under high-altitude conditions.

During spring-summer, adaptive cellular immunity in the HA group remains persistently suppressed, though some recovery in Th cell activation signaling is evident. The HA group showed upregulation of genes promoting Th1 differentiation (*HLX*) from autumn-winter to spring-summer, indicating a shift toward Th1 polarization [61]. However, this recovery is constrained by persistent upregulation of immune checkpoint molecules (*ADORA2A*) and other inhibitory factors (*LILRB3*), which dampen T cell activation and proliferation. Additionally, genes critical for T cell proliferation (*IL15*) and cytotoxic function (*B2M*) remained lower in the HA compared to the LA, indicating incomplete restoration of T cell effector functions. This pattern suggests that Th cell activation recovery primarily manifests as upregulation of differentiation signaling pathways rather than extensive T cell proliferation and cytotoxic capacity. In contrast to the persistent suppression of cellular immunity, humoral immunity showed partial enhancement in HA. From autumn-winter to spring-summer, B cell activation, proliferation, and differentiation pathways were upregulated, with key regulators *PAX5*, *RUNX1*, and *AICDA* showing elevated expression [62,63]. *AICDA* upregulation is particularly significant as it drives antibody class-switching and affinity maturation. This preserved humoral immune capacity suggests that vaccination programs may be effective in high-altitude populations, though vaccine timing should account for the seasonal immune dynamics—spring-summer may represent a more favorable window for eliciting robust antibody responses. The Fcγ receptor-mediated phagocytosis pathway was upregulated from autumn-winter to spring-summer in the HA (*NCF1*, *CFL1*, *SPHK1*, *GSN*, and *GAB2* were upregulated). This pathway enhances antibody-dependent phagocytosis: IgG antibodies produced by B cells bind to Fcγ receptors on macrophages and neutrophils, and receptor cross-linking activates downstream signaling cascades involving PI3K, Syk, and actin remodeling, promoting phagocytosis and intracellular killing [64,65,66]. Combined with restored neutrophil function, this forms an efficient antibody-mediated pathogen clearance network, indicating enhanced antibody effector functions in spring-summer HA.

Integrating innate and adaptive immune responses, high-altitude forest musk deer exhibited a constrained immune pattern during spring-summer characterized by energy-metabolism trade-offs: peripheral blood myeloid cells and T cells maintain limited function to sustain low inflammation and reduce excessive energy expenditure; enhanced expression of transendothelial migration pathway components attempts to maximize recruitment of limited immune cells to infection sites, though effectiveness remains constrained by reduced circulating cell numbers; B cells maintain capacity for primary antibody production, particularly T-independent responses, which synergizes with partially recovered neutrophils via the Fcγ receptor-phagocytosis axis to clear opsonized pathogens. This pattern represents an energy-conservation strategy typical of resource-limited environments, prioritizing immune efficiency over maximal immune capacity. However, persistent immune vulnerabilities require attention. Despite functional recovery, neutrophil activity remains weaker than in low-altitude groups, elevating susceptibility to pyogenic bacterial infections; T-cell proliferation and CTL responses are constrained, potentially compromising clearance of intracellular pathogens and viral infections; IgG class switching is reduced, limiting high-affinity antibody production and effector functions. Under captive conditions, management interventions are required: optimizing nutritional support to enhance immune cell production and function, implementing vaccination programs that account for limited T-dependent responses, establishing enhanced disease surveillance for early infection detection, and maintaining strict biosecurity to reduce pathogen exposure.

#### 4.1.3. Seasonal Immune Dynamics

The seasonal comparison within the high-altitude group reveals a distinctive pattern of immune remodeling that reflects adaptive responses to changing environmental conditions. From autumn-winter to spring-summer, high-altitude forest musk deer exhibit a transition from comprehensive immune suppression to selective functional recovery, representing a dynamic energy allocation strategy responsive to seasonal environmental fluctuations.

Several environmental factors likely drive these seasonal immune dynamics. First, ambient temperature variations between seasons directly influence thermoregulatory energy demands: the harsh cold of autumn-winter necessitates substantial energy allocation to thermogenesis, leaving limited resources for immune function, whereas milder spring-summer temperatures reduce this metabolic burden and permit partial immune recovery. Second, food availability and nutritional quality vary seasonally in high-altitude environments: the scarcity of quality forage during autumn-winter further constrains energy budgets, while improved vegetation quality and abundance during spring-summer provides enhanced nutritional support for immune cell production and function.

Our findings reveal that forest musk deer do not simply follow a linear suppression-recovery pattern, but rather exhibit selective immune remodeling. Notably, NK cell function remains elevated year-round compared to low-altitude populations and shows further enhancement during spring-summer, while T cell responses remain persistently suppressed across both seasons. This differential recovery pattern suggests that energy allocation to immune components is prioritized based on cost-efficiency rather than uniformly restored when resources become available. The preferential maintenance and enhancement of NK cells—which provide rapid, antigen-independent cytotoxicity with minimal metabolic demands—over energy-intensive adaptive immunity represents an optimized strategy for maintaining essential immune surveillance under persistent resource constraints.

In the context of existing high-altitude adaptation research, our findings reveal several unexpected observations. First, the selective enhancement of NK cell function despite comprehensive immune suppression was unanticipated. Unlike the broadly activated immune responses observed in yak compared to cattle [46], forest musk deer exhibit a more nuanced pattern where specific immune components are strategically preserved. This selective preservation of energy-efficient innate immunity represents a previously unrecognized adaptive strategy prioritizing metabolic economy under resource constraints.

Second, the asymmetric recovery of innate versus adaptive immunity during spring-summer contrasts with expectations of uniform immune recovery when environmental conditions improve. While humoral immunity (*PAX5*, *RUNX1*, *AICDA*) and neutrophil-mediated responses showed significant recovery, T cell-mediated cellular immunity remained suppressed, suggesting differential prioritization of immune components based on energy cost-efficiency rather than equal restoration.

Third, the enhanced B cell function despite T helper cell suppression suggests potential reliance on T-independent antibody responses—a finding with important implications for vaccination strategies in high-altitude populations. Collectively, these findings demonstrate that high-altitude immune adaptation in forest musk deer involves sophisticated selective mechanisms rather than simple broad-spectrum suppression, offering new perspectives on immune-metabolic trade-offs in energy-limited environments.

### 4.2. Adaptive Features of Energy Metabolism in High-Altitude Forest Musk Deer

Energy metabolism underpins immune function. High-altitude environments exert dual stressors of hypoxia and cold temperatures, profoundly altering metabolic patterns. Transcriptomic analysis in this study revealed seasonal metabolic change in high-altitude forest musk deer: transitioning from a “hypoxic energy-efficient” mode in autumn-winter to a “enhanced metabolism and reserve accumulation” mode in spring-summer. This metabolic reprogramming provides the foundation for seasonal regulation of immune function.

During autumn-winter, the HA exhibited significant metabolic differences compared to the LA, characterized by prioritizing anaerobic pathways and suppressing energy-intensive processes. Glucose metabolism significantly enhanced the anaerobic glycolysis pathway (upregulation of genes related to lactate/pyruvate metabolism) while simultaneously inhibiting aerobic oxidation pathways. Although glycolysis has low ATP production efficiency, its advantage lies in its oxygen-independent nature and rapid reaction speed, making it suited for high-altitude environments. Lipid metabolism exhibited overall suppression of lipid synthesis pathways (downregulation of fatty acid/triglyceride synthesis genes) and enhanced fat mobilization (upregulation of lipase activity genes). Fatty acid synthesis is highly energy-intensive, requiring 7 ATP and 14 NADPH molecules per palmitate molecule, making its suppression consistent with an energy-conserving strategy. Fat mobilization releases free fatty acids (FFAs) as an alternative energy source, offering high energy density and glucose conservation [67]. It also supports non-shivering thermogenesis in brown adipose tissue (BAT), where fatty acid oxidation is uncoupled from ATP synthesis by UCP1, converting the energy into heat to maintain body temperature in cold environments [68]. Protein and amino acid metabolism exhibited dual regulation: protein degradation is suppressed (via downregulation of peptidase activity genes) to prevent excessive skeletal muscle breakdown during energy-limited periods, as inflammatory conditions can activate proteolytic pathways leading to muscle wasting [69]; specific amino acid metabolism is upregulated (histidine/glutamine) to support gluconeogenesis for maintaining blood glucose stability [70,71] and enhance glutathione (GSH) synthesis for antioxidant defense [72]. Metabolic allocation during autumn-winter prioritizes energy demands for essential life functions like thermoregulation while reducing energy expenditure on immunity.

The HA in spring-summer exhibited enhanced metabolic activity and energy reserves, promoting protein synthesis and lipid storage, thereby providing a material foundation for partial restoration of immune function. From autumn-winter to spring-summer, the HA exhibited significantly upregulated protein biosynthesis processes (e.g., upregulation of peptide biosynthesis-related genes) alongside sustained inhibition of protein degradation (downregulation of pathways including negative regulation of proteolysis and peptidase activity). This enhanced protein synthesis provides crucial material foundations for biological processes such as immune cell proliferation and differentiation, production of immune-related proteins, and tissue repair and growth. Multiple lipid synthesis-related pathways, such as unsaturated fatty acid and triglyceride biosynthesis, were upregulated. This is crucial for cell membrane biosynthesis and the generation of lipid signaling molecules, while also enhancing energy reserves. From autumn-winter to spring-summer, the HA showed no significant differences in glucose metabolism pathways, suggesting that glucose metabolism may be maintained at a baseline level throughout the year. The sustained activity of glycolysis likely represents the primary energy acquisition pathway for high-altitude forest musk deer.

Compared to the LA, the HA during spring-summer exhibited upregulation of adipocyte differentiation pathways (white adipocyte differentiation, brown adipocyte differentiation, and adipocyte proliferation regulation). Enhanced white adipocyte differentiation supports long-term energy storage [73], while upregulation of brown adipocyte differentiation indicates increased thermogenic capacity. Upregulation of adipocyte proliferation regulation suggests enhanced capacity for adipose tissue expansion, providing a foundation for energy accumulation that can be mobilized during autumn-winter. Compared to the LA, the high-altitude group during spring-summer exhibited downregulation of positive regulation in lipase and phospholipase activity, along with upregulation of negative regulation in proteolysis and peptidase activity (i.e., enhanced protein degradation inhibition). Lipase catalyzes triglyceride hydrolysis to release FFAs; its restricted activity reduces lipolysis, preserving energy reserves [74]. Phospholipase hydrolyzes membrane phospholipids to release inflammatory mediator precursors like arachidonic acid; its inhibition reduces pro-inflammatory signaling, maintaining a low-inflammation state [75]. Enhanced protein degradation inhibition ensures adequate availability of essential immune molecules (antibodies, cytokines) and structural proteins during spring-summer immune function restoration.

### 4.3. Implications for Captive Breeding and Health Management

The seasonal immune-metabolic dynamics characterized in this study have direct and actionable implications for health management and breeding practices in high-altitude captive forest musk deer populations.

Seasonal health management strategies. (1) Disease risk assessment: Autumn-winter represents a period of heightened disease vulnerability due to comprehensive immune suppression. Enhanced biosecurity measures, reduced animal handling to minimize stress-induced immunosuppression, and vigilant health monitoring with early intervention protocols are recommended during this period. (2) Nutritional intervention: The energy-immune trade-off suggests that optimizing nutritional support—particularly during autumn-winter—may help mitigate immune suppression by reducing metabolic constraints. High-energy feed supplementation with adequate protein content should be considered to support both thermogenesis and baseline immune function. (3) Vaccination timing: Given the partial recovery of humoral immunity during spring-summer, this period may represent an optimal window for vaccination programs.

Breeding practice adjustments. The seasonal immune-metabolic patterns have important implications for reproductive management: (1) Breeding season considerations: The natural breeding season of forest musk deer coincides with the period of maximum immune suppression. Breeding animals should receive enhanced nutritional support and reduced handling during this period to minimize additional physiological stress. (2) Pre-breeding health optimization: Health assessments and any necessary treatments should be conducted during spring-summer when immune function is more robust, rather than during the immunocompromised autumn-winter period. (3) Post-partum care timing: Fawns born in spring-summer benefit from the relatively enhanced maternal immune status; however, attention should be given to the transition into autumn-winter when both dams and young fawns face increasing immune constraints.

### 4.4. Limitations and Future Directions

Several limitations should be acknowledged. First, the modest sample size, particularly for autumn-winter high-altitude group (*n* = 4), was constrained by limited animal availability and harsh sampling conditions. While we employed methods optimized for small samples (edgeR with TMM normalization) and stringent thresholds, larger-scale studies would provide greater statistical power. Second, blood transcriptomics primarily reflects circulating immune cells; tissue-specific analyses and multi-omics integration would offer deeper insights. Third, captive populations may not fully represent wild populations experiencing additional ecological pressures. Despite these limitations, our study provides the first comprehensive characterization of seasonal immune dynamics in high-altitude forest musk deer, establishing a foundation for future research with larger sample sizes and multi-omics approaches.

## 5. Conclusions

This study reveals three key findings regarding immune-metabolic adaptation in high-altitude captive forest musk deer. First, high-altitude populations exhibit comprehensive immune suppression during autumn-winter, with selective preservation of NK cell function representing an energy-efficient defense strategy. Second, spring-summer shows asymmetric immune recovery: innate immunity and humoral responses partially recover while T cell-mediated cellular immunity remains suppressed, indicating prioritization based on energy cost-efficiency. Third, metabolic reprogramming shifts from energy conservation in autumn-winter to enhanced anabolism and reserve accumulation in spring-summer, providing the foundation for seasonal immune dynamics. These findings demonstrate that high-altitude immune adaptation involves sophisticated selective mechanisms rather than simple broad-spectrum suppression. The persistent immune constraints, particularly during autumn-winter, have direct implications for disease prevention and vaccination timing in captive breeding programs. Several limitations should be noted, including modest sample sizes and the use of blood transcriptomics alone; future studies with larger cohorts and multi-omics approaches would strengthen these findings.

## Figures and Tables

**Table 1:** Details of RNA-seq libraries from 33 blood samples in this study.

Sample ID	Organ	Sex	Age	Sampling Season	Group	Read Length (bp)	Layout	Aligned Reads (%)
SX19008	Blood	Male	5	Autumn-Winter	LA	150	Paired	97.01%
SX21101	Blood	Male	3	Autumn-Winter	LA	150	Paired	95.95%
SX21265	Blood	Male	3	Autumn-Winter	LA	150	Paired	96.95%
SX22362	Blood	Male	5	Autumn-Winter	LA	150	Paired	96.48%
SX21070	Blood	Female	3	Autumn-Winter	LA	150	Paired	96.45%
SX19201	Blood	Female	5	Autumn-Winter	LA	150	Paired	96.40%
SX22371	Blood	Female	5	Autumn-Winter	LA	150	Paired	96.17%
SX22370	Blood	Female	5	Autumn-Winter	LA	150	Paired	95.91%
T-24940-5	Blood	Male	5	Autumn-Winter	HA	150	Paired	96.01%
T-031-3	Blood	Male	3	Autumn-Winter	HA	150	Paired	95.88%
T-002-5	Blood	Male	5	Autumn-Winter	HA	150	Paired	95.80%
T-049-3	Blood	Male	3	Autumn-Winter	HA	150	Paired	96.76%
SX16520	Blood	Male	5	Spring-Summer	LA	150	Paired	97.76%
SX17008	Blood	Male	3	Spring-Summer	LA	150	Paired	98.05%
SX17096	Blood	Female	5	Spring-Summer	LA	150	Paired	98.14%
SX17215	Blood	Male	5	Spring-Summer	LA	150	Paired	98.29%
SX17273	Blood	Female	3	Spring-Summer	LA	150	Paired	98.19%
SX17292	Blood	Male	5	Spring-Summer	LA	150	Paired	97.76%
SX17337	Blood	Female	3	Spring-Summer	LA	150	Paired	97.94%
SX20088	Blood	Female	3	Spring-Summer	LA	150	Paired	98.16%
SX21103	Blood	Male	3	Spring-Summer	LA	150	Paired	98.05%
BB035	Blood	Female	3	Spring-Summer	HA	150	Paired	98.17%
BB037	Blood	Male	3	Spring-Summer	HA	150	Paired	96.72%
BB046	Blood	Male	3	Spring-Summer	HA	150	Paired	98.22%
BB24819	Blood	Male	6	Spring-Summer	HA	150	Paired	97.67%
BB24931	Blood	Female	6	Spring-Summer	HA	150	Paired	97.94%
BB24938	Blood	Male	6	Spring-Summer	HA	150	Paired	96.85%
BB24940	Blood	Male	6	Spring-Summer	HA	150	Paired	97.89%
BB31453	Blood	Male	6	Spring-Summer	HA	150	Paired	98.46%
BB33161	Blood	Male	6	Spring-Summer	HA	150	Paired	98.01%
BB54849	Blood	Male	3	Spring-Summer	HA	150	Paired	98.23%
BB54866	Blood	Male	3	Spring-Summer	HA	150	Paired	97.71%
BB54878	Blood	Male	3	Spring-Summer	HA	150	Paired	98.37%

Abbreviations: HA, high altitude (~3900 m); LA, low altitude (~1450 m); bp, base pairs.

**Table 2:** Quality evaluation statistics of RNA-seq data from forest musk deer.

Sample ID	Group	Sampling Season	Raw Reads	Clean Reads	Cleaning Rate (%)	Q20 (%)	Q30 (%)	GC Content (%)	Alignment Rate (%)
SX19008	LA	Autumn-Winter	68,331,458	67,572,988	98.89	98.02	94.48	51.80	97.01%
SX21101	LA	Autumn-Winter	54,823,128	54,133,828	98.74	97.60	93.72	53.44	95.95%
SX21265	LA	Autumn-Winter	41,502,752	41,059,058	98.93	97.94	94.58	53.19	96.95%
SX22362	LA	Autumn-Winter	46,336,874	45,661,340	98.54	97.41	93.29	52.35	96.48%
SX21070	LA	Autumn-Winter	49,603,532	48,983,822	98.75	98.02	94.66	53.27	96.45%
SX19201	LA	Autumn-Winter	50,758,582	50,060,358	98.62	97.50	93.50	52.30	96.40%
SX22371	LA	Autumn-Winter	820,164,894	802,627,330	97.86	96.73	91.69	51.48	96.17%
SX22370	LA	Autumn-Winter	45,196,232	44,573,762	98.62	97.65	93.86	52.61	95.91%
T-24940-5	HA	Autumn-Winter	60,799,270	59,983,050	98.66	98.06	94.53	53.13	96.01%
T-031-3	HA	Autumn-Winter	59,159,962	58,339,592	98.61	97.96	94.34	52.49	95.88%
T-002-5	HA	Autumn-Winter	58,372,462	57,651,532	98.76	98.11	94.66	53.32	95.80%
T-049-3	HA	Autumn-Winter	60,930,064	59,982,620	98.45	98.40	95.34	53.73	96.76%
SX16520	LA	Spring-Summer	43,108,044	41,745,932	96.84	97.38	93.30	51.03	97.76%
SX17008	LA	Spring-Summer	55,487,562	54,603,680	98.41	97.37	93.16	49.81	98.05%
SX17096	LA	Spring-Summer	58,791,770	58,261,426	99.10	97.29	93.02	49.84	98.14%
SX17215	LA	Spring-Summer	44,678,976	43,959,018	98.39	97.31	93.10	50.99	98.29%
SX17273	LA	Spring-Summer	47,271,884	46,781,814	98.96	97.25	92.96	48.72	98.19%
SX17292	LA	Spring-Summer	50,070,076	49,508,964	98.88	97.35	93.15	50.02	97.76%
SX17337	LA	Spring-Summer	57,952,334	57,383,428	99.02	97.26	92.97	49.15	97.94%
SX20088	LA	Spring-Summer	47,874,450	47,183,824	98.56	97.11	92.77	50.55	98.16%
SX21103	LA	Spring-Summer	45,138,214	44,400,380	98.37	97.04	92.64	47.88	98.05%
BB035	HA	Spring-Summer	53,238,930	52,037,320	97.74	97.82	94.27	50.99	98.17%
BB037	HA	Spring-Summer	39,232,034	38,580,984	98.34	97.68	94.10	51.40	96.72%
BB046	HA	Spring-Summer	48,690,754	47,900,884	98.38	97.82	94.28	51.23	98.22%
BB24819	HA	Spring-Summer	61,203,202	60,633,830	99.07	97.71	93.94	49.49	97.67%
BB24931	HA	Spring-Summer	51,425,108	50,726,218	98.64	97.94	94.42	49.31	97.94%
BB24938	HA	Spring-Summer	41,941,464	41,210,594	98.26	97.58	93.90	49.21	96.85%
BB24940	HA	Spring-Summer	45,523,120	44,627,644	98.03	97.58	93.87	50.22	97.89%
BB31453	HA	Spring-Summer	61,096,524	60,554,168	99.11	98.04	94.71	51.25	98.46%
BB33161	HA	Spring-Summer	46,917,198	45,927,702	97.89	97.78	94.24	50.23	98.01%
BB54849	HA	Spring-Summer	58,574,812	57,999,440	99.02	97.92	94.44	50.33	98.23%
BB54866	HA	Spring-Summer	43,063,594	42,237,312	98.08	97.58	93.96	50.35	97.71%
BB54878	HA	Spring-Summer	46,511,224	45,756,470	98.38	97.82	94.36	51.13	98.37%

## Data Availability

The high-throughput sequencing data from this study have been submitted to the NCBI with the project accession PRJNA1346646.

## Supplementary Materials

The following supporting information can be downloaded at: https://www.mdpi.com/article/10.3390/ani15233501/s1, Table S1: Top 10 DEGs for autumn-winter HA vs. LA (ranked by FDR); Table S2: Top 10 DEGs for spring-summer HA vs. LA (ranked by FDR); Table S3: Top 10 DEGs for HA spring-summer vs. autumn-winter (ranked by FDR); Table S4: Summary of bioinformatics analysis workflow.

## References

[1] Burtscher J., Pasha Q., Chanana N., Millet G.P., Burtscher M., Strasser B. (2023). Immune Consequences of Exercise in Hypoxia: A Narrative Review. J. Sport Health Sci..

[2] Krzywinska E., Stockmann C. (2018). Hypoxia, Metabolism and Immune Cell Function. Biomedicines.

[3] Reddan B., Cummins E.P. (2025). The Regulation of Cell Metabolism by Hypoxia and Hypercapnia. J. Biol. Chem..

[4] Nord A., Hegemann A., Folkow L.P. (2020). Reduced Immune Responsiveness Contributes to Winter Energy Conservation in an Arctic Bird. J. Exp. Biol..

[5] Vialard F., Olivier M. (2020). Thermoneutrality and Immunity: How Does Cold Stress Affect Disease?. Front. Immunol..

[6] Watson H., Nilsson J.-Å., Nilsson J.F. (2023). Thermoregulatory Costs of the Innate Immune Response Are Modulated by Winter Food Availability in a Small Passerine. J. Anim. Ecol..

[7] Yan Z., Yang J., Wei W.-T., Zhou M.-L., Mo D.-X., Wan X., Ma R., Wu M.-M., Huang J.-H., Liu Y.-J. (2024). A Time-Resolved Multi-Omics Atlas of Transcriptional Regulation in Response to High-Altitude Hypoxia across Whole-Body Tissues. Nat. Commun..

[8] Pham K., Vargas A., Frost S., Shah S., Heinrich E.C. (2024). Changes in Immune Cell Populations during Acclimatization to High Altitude. Physiol. Rep..

[9] Witt K.E., Huerta-Sánchez E. (2019). Convergent Evolution in Human and Domesticate Adaptation to High-Altitude Environments. Philos. Trans. R. Soc. B Biol. Sci..

[10] Zhao P., Li S., He Z., Ma X. (2024). Physiological and Genetic Basis of High-Altitude Indigenous Animals’ Adaptation to Hypoxic Environments. Animals.

[11] Wang B., He J., Cui Y., Yu S., Zhang H., Wei P., Zhang Q. (2024). The HIF-1α/EGF/EGFR Signaling Pathway Facilitates the Proliferation of Yak Alveolar Type II Epithelial Cells in Hypoxic Conditions. Int. J. Mol. Sci..

[12] Mishra K.P., Ganju L., Singh S.B. (2015). Hypoxia Modulates Innate Immune Factors: A Review. Int. Immunopharmacol..

[13] Xi L., Xia Z., Cai F., Zhao S., Chen Z., Zhuo L., Yang G., Yan Q., Zheng C., Wang H. (2016). Distribution and antibiotic resistance analysis of *Pseudomonas aeruginosa* in captive musk deer breeding farms. Zhejiang Agric. J..

[14] Xi L., Chen Z., Song H., Ren L., Wen J., Huang J., Yang R., Yang G., Wang H., Yan Q. (2018). Isolation and identification of *Pseudomonas aeruginosa* from captive musk deer, and analysis of its drug resistance and pathogenic characteristics. Microbiol. China.

[15] Hu D., Hou Z., Deng C., Chen X., Wu J., Chen L., Chen Q., Yue B., Zhang X. (2021). Study on the genetic diversity of musk deer in the Baishuihe National Nature Reserve of Sichuan based on microsatellites and mitochondria. Sichuan J. Zool..

[16] Yuan Y., Li J., Zhang A., Lin J., Chu Y., Wang X., Zhao K. (2020). Study on the interaction mechanism between *Corynebacterium pseudotuberculosis* and *Pseudomonas aeruginosa* in musk deer with purulent disease. Sichuan J. Zool..

[17] Xu Z., Xu H. (2003). Population Characteristics and Fawn Survival in Musk Deer (*Moshus moschiferus*). Acta Theriol. Sin..

[18] LI Y., Zhang T., Shi M., Zhang B., Hu X., Xu S., Ding J., Liu S., Hu D., Rubenstein D. (2021). Characterization of Intestinal Microbiota and Fecal Cortisol, T3, and IgA in Forest Musk Deer (*Moschus berezovskii*) from Birth to Weaning. Integr. Zool..

[19] Sun X., Cai R., Jin X., Shafer A., Hu X., Yang S., Li Y., Lei Q., Liu S., Hu D. (2018). Blood Transcriptomics of Captive Forest Musk Deer (*Moschus berezovskii*) and Possible Associations with the Immune Response to Abscesses. Sci. Rep..

[20] Tang J., Suo L., Li F., Yang C., Bian K., Wang Y. (2022). ITRAQ-Based Quantitative Proteomics Analysis of Forest Musk Deer with Pneumonia. Front. Vet. Sci..

[21] Chen B., Li D., Ran B., Zhang P., Wang T. (2022). Key miRNAs and Genes in the High-Altitude Adaptation of Tibetan Chickens. Front. Vet. Sci..

[22] Qi X., Zhang Q., He Y., Yang L., Zhang X., Shi P., Yang L., Liu Z., Zhang F., Liu F. (2018). The Transcriptomic Landscape of Yaks Reveals Molecular Pathways for High Altitude Adaptation. Genome Biol. Evol..

[23] Verma P., Sharma A., Sodhi M., Thakur K., Kataria R.S., Niranjan S.K., Bharti V.K., Kumar P., Giri A., Kalia S. (2018). Transcriptome Analysis of Circulating PBMCs to Understand Mechanism of High Altitude Adaptation in Native Cattle of Ladakh Region. Sci. Rep..

[24] Goronzy J.J., Weyand C.M. (2013). Understanding Immunosenescence to Improve Responses to Vaccines. Nat. Immunol..

[25] Müller L., Di Benedetto S., Pawelec G. (2019). The Immune System and Its Dysregulation with Aging. Subcell. Biochem..

[26] Feng H., Wang L., Cao F., Ma J., Tang J., Feng C., Su Z. (2023). Forest Musk Deer (*Moschus berezovskii*) in China: Research and Protection. J. Vertebr. Biol..

[27] Wang J., Zheng Q., Xia C., Li Y., Zhou M., Sheng Y., Weladji R., Meng X. (2021). Seasonal Reproduction of Northernmost Endangered Forest Musk Deer (*Moschus berezovskii*) in China and the Synchronization with Climatic Conditions. Czech J. Anim. Sci..

[28] Patel R.K., Jain M. (2012). NGS QC Toolkit: A Toolkit for Quality Control of next Generation Sequencing Data. PLoS ONE.

[29] Kim D., Paggi J.M., Park C., Bennett C., Salzberg S.L. (2019). Graph-Based Genome Alignment and Genotyping with HISAT2 and HISAT-Genotype. Nat. Biotechnol..

[30] Wang T., Yang M., Shi X., Tian S., Li Y., Xie W., Zou Z., Leng D., Zhang M., Zheng C. (2025). Multiomics Analysis Provides Insights into Musk Secretion in Muskrat and Musk Deer. Gigascience.

[31] Li H., Handsaker B., Wysoker A., Fennell T., Ruan J., Homer N., Marth G., Abecasis G., Durbin R., 1000 Genome Project Data Processing Subgroup (2009). The Sequence Alignment/Map Format and SAMtools. Bioinformatics.

[32] Liao Y., Smyth G.K., Shi W. (2014). featureCounts: An Efficient General Purpose Program for Assigning Sequence Reads to Genomic Features. Bioinformatics.

[33] Robinson M.D., Oshlack A. (2010). A Scaling Normalization Method for Differential Expression Analysis of RNA-Seq Data. Genome Biol..

[34] Valero-Mora P.M. (2010). Ggplot2: Elegant Graphics for Data Analysis. J. Stat. Softw..

[35] Schurch N.J., Schofield P., Gierliński M., Cole C., Sherstnev A., Singh V., Wrobel N., Gharbi K., Simpson G.G., Owen-Hughes T. (2016). How Many Biological Replicates Are Needed in an RNA-Seq Experiment and Which Differential Expression Tool Should You Use?. RNA.

[36] Zhou X., Lindsay H., Robinson M.D. (2014). Robustly Detecting Differential Expression in RNA Sequencing Data Using Observation Weights. Nucleic Acids Res..

[37] Benjamini Y., Hochberg Y. (1995). Controlling the False Discovery Rate: A Practical and Powerful Approach to Multiple Testing. J. R. Stat. Soc. Ser. B Methodol..

[38] Love M.I., Huber W., Anders S. (2014). Moderated Estimation of Fold Change and Dispersion for RNA-Seq Data with DESeq2. Genome Biol..

[39] Conesa A., Madrigal P., Tarazona S., Gomez-Cabrero D., Cervera A., McPherson A., Szcześniak M.W., Gaffney D.J., Elo L.L., Zhang X. (2016). A Survey of Best Practices for RNA-Seq Data Analysis. Genome Biol..

[40] Yu G. (2018). clusterProfiler: An Universal Enrichment Tool for Functional and Comparative Study. bioRxiv.

[41] Szklarczyk D., Franceschini A., Wyder S., Forslund K., Heller D., Huerta-Cepas J., Simonovic M., Roth A., Santos A., Tsafou K.P. (2015). STRING V10: Protein-Protein Interaction Networks, Integrated over the Tree of Life. Nucleic Acids Res..

[42] Shannon P., Markiel A., Ozier O., Baliga N.S., Wang J.T., Ramage D., Amin N., Schwikowski B., Ideker T. (2003). Cytoscape: A Software Environment for Integrated Models of Biomolecular Interaction Networks. Genome Res..

[43] Lochmiller R.L., Deerenberg C. (2000). Trade-Offs in Evolutionary Immunology: Just What Is the Cost of Immunity?. Oikos.

[44] Qiu Q., Zhang G., Ma T., Qian W., Wang J., Ye Z., Cao C., Hu Q., Kim J., Larkin D.M. (2012). The Yak Genome and Adaptation to Life at High Altitude. Nat. Genet..

[45] Ge R.-L., Cai Q., Shen Y.-Y., San A., Ma L., Zhang Y., Yi X., Chen Y., Yang L., Huang Y. (2013). Draft Genome Sequence of the Tibetan Antelope. Nat. Commun..

[46] Wang K., Yang Y., Wang L., Ma T., Shang H., Ding L., Han J., Qiu Q. (2016). Different Gene Expressions between Cattle and Yak Provide Insights into High-Altitude Adaptation. Anim. Genet..

[47] Ricklin D., Hajishengallis G., Yang K., Lambris J.D. (2010). Complement: A Key System for Immune Surveillance and Homeostasis. Nat. Immunol..

[48] Onai N., Obata-Onai A., Schmid M.A., Ohteki T., Jarrossay D., Manz M.G. (2007). Identification of Clonogenic Common Flt3^+^M-CSFR^+^ Plasmacytoid and Conventional Dendritic Cell Progenitors in Mouse Bone Marrow. Nat. Immunol..

[49] Pflanz S., Timans J.C., Cheung J., Rosales R., Kanzler H., Gilbert J., Hibbert L., Churakova T., Travis M., Vaisberg E. (2002). IL-27, a Heterodimeric Cytokine Composed of EBI3 and P28 Protein, Induces Proliferation of Naive CD4^+^ T Cells. Immunity.

[50] Carroll M.C. (2004). The Complement System in Regulation of Adaptive Immunity. Nat. Immunol..

[51] Vivier E., Raulet D.H., Moretta A., Caligiuri M.A., Zitvogel L., Lanier L.L., Yokoyama W.M., Ugolini S. (2011). Innate or Adaptive Immunity? The Example of Natural Killer Cells. Science.

[52] Doumas S., Kolokotronis A., Stefanopoulos P. (2005). Anti-Inflammatory and Antimicrobial Roles of Secretory Leukocyte Protease Inhibitor. Infect. Immun..

[53] Sallenave J.-M. (2010). Secretory Leukocyte Protease Inhibitor and Elafin/Trappin-2: Versatile Mucosal Antimicrobials and Regulators of Immunity. Am. J. Respir. Cell Mol. Biol..

[54] Bedard K., Krause K.-H. (2007). The NOX Family of ROS-Generating NADPH Oxidases: Physiology and Pathophysiology. Physiol. Rev..

[55] Brinkmann V., Reichard U., Goosmann C., Fauler B., Uhlemann Y., Weiss D.S., Weinrauch Y., Zychlinsky A. (2004). Neutrophil Extracellular Traps Kill Bacteria. Science.

[56] Belaaouaj A.A., Kim K.S., Shapiro S.D. (2000). Degradation of Outer Membrane Protein a in *Escherichia coli* Killing by Neutrophil Elastase. Science.

[57] Weinrauch Y., Drujan D., Shapiro S.D., Weiss J., Zychlinsky A. (2002). Neutrophil Elastase Targets Virulence Factors of Enterobacteria. Nature.

[58] Yvan-Charvet L., Ng L.G. (2019). Granulopoiesis and Neutrophil Homeostasis: A Metabolic, Daily Balancing Act. Trends Immunol..

[59] Sun L., Wu J., Du F., Chen X., Chen Z.J. (2013). Cyclic GMP-AMP Synthase Is a Cytosolic DNA Sensor That Activates the Type I Interferon Pathway. Science.

[60] Pipkin M.E., Rao A., Lichtenheld M.G. (2010). The Transcriptional Control of the Perforin Locus. Immunol. Rev..

[61] Mullen A.C., Hutchins A.S., High F.A., Lee H.W., Sykes K.J., Chodosh L.A., Reiner S.L. (2002). Hlx Is Induced by and Genetically Interacts with T-Bet to Promote Heritable T_H_1 Gene Induction. Nat. Immunol..

[62] Nutt S.L., Heavey B., Rolink A.G., Busslinger M. (1999). Commitment to the B-Lymphoid Lineage Depends on the Transcription Factor Pax5. Nature.

[63] Muramatsu M., Kinoshita K., Fagarasan S., Yamada S., Shinkai Y., Honjo T. (2000). Class Switch Recombination and Hypermutation Require Activation-Induced Cytidine Deaminase (AID), a Potential RNA Editing Enzyme. Cell.

[64] Aderem A., Underhill D.M. (1999). Mechanisms of Phagocytosis in Macrophages. Annu. Rev. Immunol..

[65] Crowley M.T., Costello P.S., Fitzer-Attas C.J., Turner M., Meng F., Lowell C., Tybulewicz V.L., DeFranco A.L. (1997). A Critical Role for Syk in Signal Transduction and Phagocytosis Mediated by Fcgamma Receptors on Macrophages. J. Exp. Med..

[66] Nimmerjahn F., Ravetch J.V. (2008). Fcgamma Receptors as Regulators of Immune Responses. Nat. Rev. Immunol..

[67] Frayn K.N. (2013). Metabolic Regulation: A Human Perspective.

[68] Cannon B., Nedergaard J.A.N. (2004). Brown Adipose Tissue: Function and Physiological Significance. Physiol. Rev..

[69] Lecker S.H., Goldberg A.L., Mitch W.E. (2006). Protein Degradation by the Ubiquitin–Proteasome Pathway in Normal and Disease States. J. Am. Soc. Nephrol..

[70] Newsholme P., Lima M.M.R., Procópio J., Pithon-Curi T.C., Bazotte R.B., Curi R. (2003). Glutamine and Glutamate as Vital Metabolites. Braz. J. Med. Biol. Res..

[71] Yang T., Cheng Z., Jiang M., Ma X., Datsomor O., Zhao G., Zhan K. (2021). Histidine Promotes the Glucose Synthesis through Activation of the Gluconeogenic Pathway in Bovine Hepatocytes. Animals.

[72] Lu S.C. (2013). Glutathione Synthesis. Biochim. Biophys. Acta BBA-Gen. Subj..

[73] Rosen E.D., Spiegelman B.M. (2014). What We Talk about When We Talk about Fat. Cell.

[74] Duncan R.E., Ahmadian M., Jaworski K., Sarkadi-Nagy E., Sul H.S. (2007). Regulation of Lipolysis in Adipocytes. Annu. Rev. Nutr..

[75] Dennis E.A., Norris P.C. (2015). Eicosanoid Storm in Infection and Inflammation. Nat. Rev. Immunol..

